# Therapeutic Potential of Resveratrol for Glioma: A Systematic Review and Meta-Analysis of Animal Model Studies

**DOI:** 10.3390/ijms242316597

**Published:** 2023-11-22

**Authors:** Ângelo Luís, Helena Marcelino, Fernanda Domingues, Luísa Pereira, José Francisco Cascalheira

**Affiliations:** 1 Centro de Investigação em Ciências da Saúde (CICS-UBI), Universidade da Beira Interior, Av. Infante D. Henrique, 6200-506 Covilhã, Portugal; helena.marcelino@ubi.pt (H.M.); fdomingues@ubi.pt (F.D.); jfcascalheira@ubi.pt (J.F.C.); 2 Departamento de Química, Faculdade de Ciências, Universidade da Beira Interior, Rua Marquês D’Ávila e Bolama, 6201-001 Covilhã, Portugal; 3 Grupo de Revisões Sistemáticas (GRUBI), Faculdade de Ciências da Saúde, Universidade da Beira Interior, Av. Infante D. Henrique, 6200-506 Covilhã, Portugal; lpereira@ubi.pt; 4 Departamento de Matemática, Faculdade de Ciências, Universidade da Beira Interior, Rua Marquês D’Ávila e Bolama, 6201-001 Covilhã, Portugal; 5 Centro de Matemática e Aplicações (CMA-UBI), Universidade da Beira Interior, Rua Marquês D’Ávila e Bolama, 6201-001 Covilhã, Portugal

**Keywords:** resveratrol, glioma, glioblastoma, animal model, systematic review, meta-analysis

## Abstract

Gliomas are aggressive malignant brain tumors, with poor prognosis despite available therapies, raising the necessity for finding new compounds with therapeutic action. Numerous preclinical investigations evaluating resveratrol’s anti-tumor impact in animal models of glioma have been reported; however, the variety of experimental circumstances and results have prevented conclusive findings about resveratrol’s effectiveness. Several databases were searched during May 2023, ten publications were identified, satisfying the inclusion criteria, that assess the effects of resveratrol in murine glioma-bearing xenografts. To determine the efficacy of resveratrol, tumor volume and animal counts were retrieved, and the data were then subjected to a random effects meta-analysis. The influence of different experimental conditions and publication bias on resveratrol efficacy were evaluated. Comparing treated to untreated groups, resveratrol administration decreased the tumor volume. Overall, the effect’s weighted standardized difference in means was −2.046 (95%CI: −3.156 to −0.936; *p*-value < 0.001). The efficacy of the treatment was observed for animals inoculated with both human glioblastoma or rat glioma cells and for different modes of resveratrol administration. The combined administration of resveratrol and temozolomide was more effective than temozolomide alone. Reducing publication bias did not change the effectiveness of resveratrol treatment. The findings suggest that resveratrol slows the development of tumors in animal glioma models.

## 1. Introduction

Gliomas are aggressive brain tumors with a poor prognosis. Among these, glioblastoma, an IV grade astrocytoma, is the most common and aggressive brain cancer, and even after combining surgical resection, radiotherapy, and chemotherapy as therapeutic strategies, glioblastoma patients’ median survival time, 14.5–16.6 months, remains low [1,2]. Among the available chemotherapeutic agents, temozolomide, a DNA-alkylating agent, and Avastin, both approved by FDA, showed some, although limited, clinical efficacy [3]. Therefore, the search for new compounds with potential therapeutic action against glioma is particularly relevant.

Resveratrol is a natural phenolic compound found in plant sources such as grapes, mulberries, peanuts, cranberries, and blueberries [4]. Several studies have shown a wide range of cellular effects of resveratrol, including antioxidant and anti-inflammatory activities as well as the ability to modulate signaling pathways, controlling cell metabolism, proliferation, autophagy, and apoptosis: all these processes play a central role in cancer initiation, promotion, and progression [5,6], suggesting a potential anti-cancer effect for resveratrol. In fact, resveratrol has been shown to act on multiple cellular targets. Resveratrol has shown the ability to: (i) increase the expression of antioxidant enzymes (SOD, catalase, and glutathione *S*-transferase), protecting cells from oxidative stress [6]; (ii) decrease STAT3, NF-kB, and COX2 activation and TNF-α expression [6,7], therefore reducing inflammation; (iii) control cell metabolism by increasing sirtuins activity; (iv) decrease activation of ERK and expression of VEGF, EGF, and of stemness markers (CD133, OCT4, and Nestin) [5] while activating MAPK phosphatase-1 (MKP-1) [6], resulting in a decrease in cell proliferation; (v) increase autophagosome formation and TP53 expression, thus promoting autophagy and apoptosis of cancer cells while producing no significant harm against normal cells [5]. Furthermore, resveratrol increased the expression of activating the receptor of natural killer cells, boosting their anti-cancer action [6].

Several pre-clinical studies have revealed the anti-cancer action of resveratrol in different tumors, including gliomas [5]. A recent epidemiologic study reported that an increase in resveratrol intake in food was associated with a lower risk of glioma [8]. Resveratrol has also been shown to reduce proliferation, induce apoptosis, decrease stemness, and increase differentiation of glioma cell lines [7,9,10,11]. Pre-clinical studies using animal models of glioma and demonstrating the antitumor effects of resveratrol have been published since the early 2000s [7,9,10]. In fact, resveratrol has been shown to reduce tumor growth and angiogenesis and increase the survival of animals bearing glioma xenografts [7,8,9,10]. Furthermore, resveratrol was able to increase the anti-tumor effect of temozolomide in an animal model of glioma [9].

In pre-clinical research aimed at enhancing human health, the use of animal models of illness is important [12]. Meta-analyses of results from animal studies are useful because they can help clarify differences between preclinical and clinical trial outcomes or enhance the design of clinical trials [13]. Despite a few recent reviews highlighting resveratrol’s anti-tumor effects on animal glioma models [14,15], a thorough meta-analysis of this research has not yet been carried out.

The goal of this work was to conduct a meta-analysis of the data gathered using animal models to evaluate the effect of resveratrol on glioma growth, which will help to clarify the therapeutic potential of this natural compound, either alone or in combination with temozolomide. The systematic review was conducted in accordance with the PRISMA (Preferred Reported Items for Systematic Reviews and Meta-Analysis) statement.

## 2. Methods

### 2.1. Search Strategy, Study Selection, and Inclusion Criteria

The search for this systematic review was performed on several databases (Pubmed, Web of Science, Scopus, and SciELO). The articles were assessed until 4 May 2023. The Boolean operator tools were used to search the electronic databases using the string: “resveratrol AND (glioma OR glioblastoma)”. The records were imported to the Mendeley Desktop version 1.19.8 software (https://www.mendeley.com/autoupdates/installers/1.19.5, accessed on 27 September 2023), which was used to remove duplicates. Then, and considering the PRISMA statement [16,17,18], the titles and abstracts of the retrieved articles were used to screen their potential for inclusion in this systematic review. Furthermore, other publications that could be added were found by reviewing the references of these articles. Lastly, a thorough analysis of the complete texts of those deemed pertinent was conducted. Two separate writers conducted the search; in the event of a disagreement, a third author was contacted. The following requirements must be met for a study to be included in this systematic review: it must assess the effects of resveratrol on a animal glioma model in comparison to a control group; it must display the tumor volume (outcome) at the start and end of resveratrol treatment; it must also specify the number of animals in each group (control and treatment); and it must include the standard deviation (SD) or standard error of the mean (SEM). When the included articles presented the results in graphics, images or figures, the software ImageJ version 1.53t (https://imagej.nih.gov/ij/download.html, accessed on 18 September 2023) was employed to determine the tumor volume.

### 2.2. Risk of Bias Assessment

The quality of the methodology of the articles included in this systematic review was evaluated by a 9-item quality checklist adapted from CAMARADES (Collaborative Approach to Meta-Analysis and Review of Animal Data in Experimental Studies) (https://www.ed.ac.uk/clinical-brain-sciences/research/camarades, accessed on 18 September 2023). Following this approach, 9 criteria were evaluated: (1) peer-review publication; (2) standardized number of tumor cells implanted; (3) randomized allocation of tumor-bearing animals to treatment and control groups; (4) blinded assessment of outcome; (5) sample size calculation performed; (6) compliance with animal welfare regulations; (7) statement of potential conflicts of interest; (8) reported the number of animals originally inoculated with tumor cells; (9) reported the explanation of any treated animals excluded from the analysis [19].

### 2.3. Data Extraction and Synthesis

The included studies were analyzed and the data (authors, year of publication, type of cells, animal model, treatment duration, resveratrol dose, and administration mode) were extracted by two independent authors to a Microsoft Excel^®^ file allowing the construction of a database. Then, the two databases were compared by a third author that analyzed and resolved any inconsistency within the extracted data. Moreover, the results of the tumor volume (initial and post-treatment) were also extracted for both control and intervention groups. These results were used to calculate the fold increase in order to perform the meta-analysis.

### 2.4. Statistical Analyses

For the considered outcome (tumor volume), an evaluation was performed on the pooled effect of the resveratrol treatment in terms of a weighted difference in means (WDM) between the change from pre- and post-treatment mean values of the intervention and control groups. Statistical analyses were undertaken using the Comprehensive Meta-Analysis software v2.0 (https://www.meta-analysis.com/index.php?cart=BNZ610089737, accessed on 18 September 2023) employing the random effects model, and the number of animals, the fold increase, and the corresponding SD of the outcome for the intervention and control groups were introduced [20]. Forest plots were generated to show the study-specific effect sizes with a 95% confidence interval (CI). The Higgins I^2^ statistic was used to measure the inconsistency amongst the results of the included studies, making it possible to classify the heterogeneity as low (25%), moderate (50%) and high (75%) [21]. Subgroup analysis was performed considering the animal model used, type of cells, intervention duration (days), administration mode, and resveratrol mean daily dose (mg/kg). This technique made it possible to investigate potential sources of variability and assess how these experimental conditions affected the extent of the resveratrol effect. To determine if there is homogeneity among the subgroups, the Chi^2^ test was also employed.

Funnel plots [22,23], Egger’s regression test [20,24], and Duval and Tweedie’s trim and fill approach [25,26] were used to evaluate the possible influence of publication bias. The trim and fill approach produces a funnel plot with the observed studies (shown as blue circles) and the required imputed studies (shown as red circles) to ensure a lack of bias. It also enables the best estimate of the unbiased pooled effect size to be derived. The sensitivity analysis was also carried out by removing each research individually to assess the stability of the outcomes.

Finally, the same methodology was applied to evaluate the effects of temozolomide when it is administered alone compared with its administration combined with resveratrol.

## 3. Results and Discussion

### 3.1. Results

#### 3.1.1. Search and Selection of Studies

**Figure 1:**
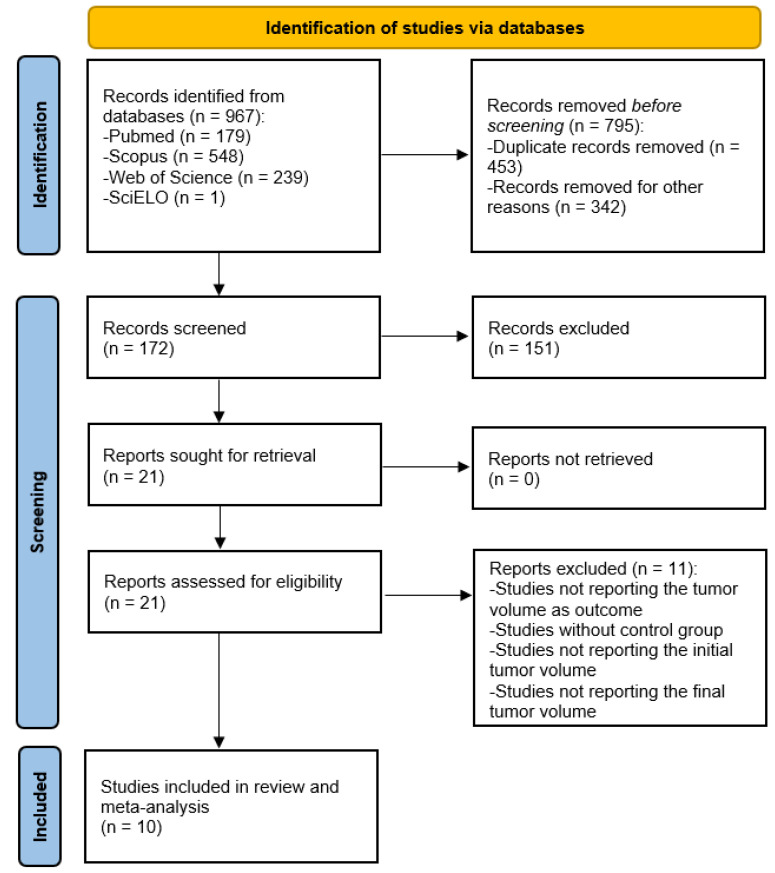
PRISMA flow diagram of the database search, study selection, and articles included in this work.

Figure 1 details the steps of the literature search for this systematic review. Initially, the electronic search allowed the identification of 967 articles with potential to be included. Then, after removing the duplicate records (453) and other papers that did not fulfil the inclusion criteria (342), for example, papers that studied the effects of resveratrol on glioblastoma in vitro or exhibited irregularities in presenting the results of the outcome under consideration, 172 papers remained. Subsequently, 151 studies were excluded after the full-text analysis, mainly because they did not present the tumor volume results. The 21 remaining eligible studies were finally checked, resulting in the exclusion of 11 due to problems in study design or in result reporting. At the end of the selection process, 10 studies were included in this systematic review with meta-analysis.

#### 3.1.2. Characteristics of the Included Studies

The characteristics of the 10 included studies in this systematic review are summarized in Table S1. The studies used both human glioblastoma and animal glioma cell lines, as well as different animal models (orthotopic or heterotopic). Different resveratrol doses, treatment duration and administration modes were employed. Such variables were also considered in the subgroup analysis.

#### 3.1.3. Risk of Bias Assessment

The studies’ quality scores, determined with the CAMARADES checklist, are presented in Table S2. The 10 included studies are peer-reviewed publications (criterion 1), and only one [9] did not report the number of tumor cells implanted in the animals (criterion 2). Concerning the randomization in the allocation of tumor-bearing animals to treatment and control groups (criterion 3), three studies did not mention the randomization process [10,11,27]. None of the included studies referred to the blinding of the outcome assessment and the sample size calculation (criteria 4 and 5). Overall, most articles presented a quality score higher than 4, indicating that they were properly conducted.

#### 3.1.4. Effects of Resveratrol on Glioma

**Figure 2:**
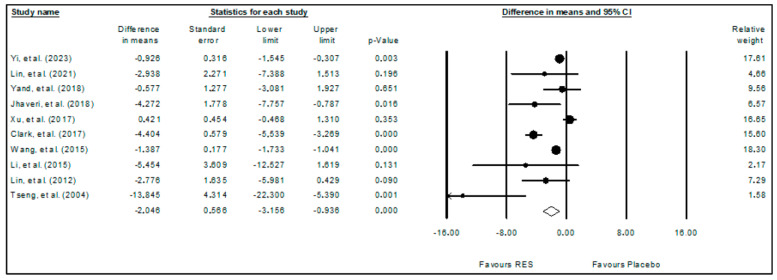
Forest plot comparing the effects of resveratrol on glioma growth. Heterogeneity: Tau^2^ = 1.720; Chi^2^ = 58.924; df = 9; *p*-value < 0.001; I^2^ = 84.726%. Test for overall effect: Z = −3.613 (*p*-value < 0.001) [9,10,11,27,28,29,30,31,32,33].

The meta-analysis results of the effects of resveratrol on glioma obtained with the random effects model are presented in the forest plot (Figure 2), considering the tumor volume (fold increase from day 1) as the analyzed outcome. The 10 included studies comprised 108 animals, and it was observed that resveratrol significantly reduced (*p*-value < 0.001) the tumor volume (WDM = −2.046; 95%CI: −3.156 to −0.936). These results indicate the potential of resveratrol to act against glioma growth. High heterogeneity among the included studies was observed (I^2^ = 84.726%).

#### 3.1.5. Subgroup and Sensitivity Analyses

A subgroup analysis was performed (Table 1) to determine the influence of the animal model used, the type of cells, the intervention duration, the administration mode, and the mean daily dose on the effects of resveratrol on glioma growth.

Regarding the animal model used, a significant reduction (*p*-value < 0.05) on the tumor volume was only observed in the heterotopic xenograft animal model (n = 8 studies; WDM = −2.179; 95%CI: −3.707 to −0.652). Only two studies were included with the orthotopic xenograft animal model. This might account for the lack of statistical significance in the reduction in glioblastoma. The animal model did not account for the observed heterogeneity (Chi^2^ = 0.047; *p*-value = 0.828).

Concerning the type of cells inoculated, a significant reduction (*p*-value < 0.05) of tumor growth was observed for both type of cells (human glioblastoma or rat glioma). Once again, the observed heterogeneity was not explained by the type of cells on which the studies were performed (Chi^2^ = 2.786; *p*-value = 0.095).

Considering the intervention duration, only for more prolonged interventions (18–30 days) was a significant reduction (*p*-value < 0.05) in tumor volume observed (n = 5 studies; WDM = −3.778; 95%CI: −5.312 to −2.243). Moreover, a significant amount of the observed heterogeneity was accounted for by the duration of the intervention (Chi^2^ = 9.778; *p*-value = 0.008). The administration mode also contributed to the heterogeneity (Chi^2^ = 9.484; *p*-value = 0.009); although a significant reduction (*p*-value < 0.05) in the tumor volume was observed for all three administration modes, intraperitoneal administration produced a smaller effect (WDM = −1.140; 95%CI: −2.079 to −0.200) than either intravenous or oral administration of resveratrol. However, this result may be considered with caution since the number of included studies employing either the intravenous or oral administration of resveratrol was only one each.

Regarding the mean daily dose of resveratrol (mg/kg), only for the higher doses (15.1–150) was a significant reduction in glioma volume observed (n = 3 studies; WDM = −3.184; 95%CI: −6.596 to −1.032). The mean daily dose of resveratrol did contribute to heterogeneity (Chi^2^ = 1.755; *p*-value = 0.635).

**Figure 3:**
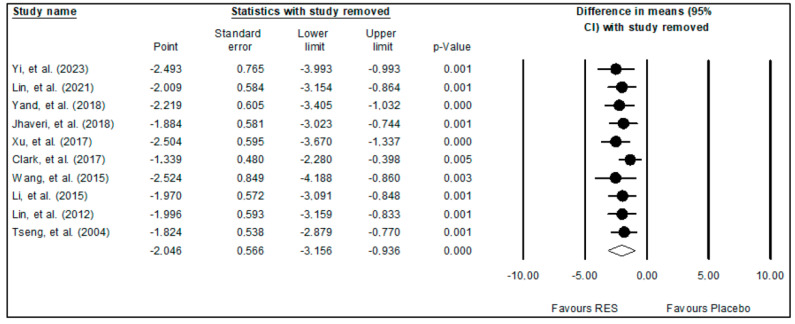
Results of sensitivity analysis [9,10,11,27,28,29,30,31,32,33].

A sensitivity analysis was also carried out to explore how the results would change if one or more studies were not included in the meta-analysis. The findings demonstrated that excluding one or a few studies did not significantly alter the pooled effects of resveratrol on glioma growth (Figure 3). Overall, the sensitivity analysis demonstrated the validity of the meta-analysis findings.

#### 3.1.6. Publication Bias

The existence of publication bias was initially studied by Egger’s regression test (Table 2). The results did not indicate the presence of publication bias (*p*-value > 0.05).

**Figure 4:**
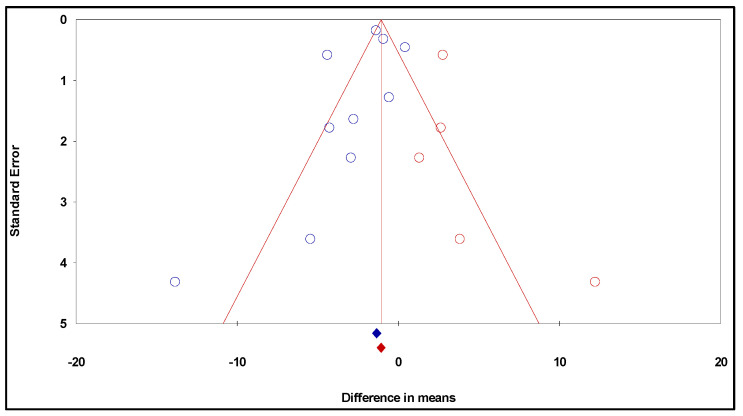
Funnel plot of standard error by difference in means (publication bias tests) of the effects of resveratrol on glioma growth: observed studies—blue circles, and the imputed studies—red circles.

Furthermore, the funnel plot was generated considering the trim and fill approach (Figure 4). This approach allowed the imputation of five additional studies on the right of the plot to adjust it to the absence of bias.

#### 3.1.7. Effects of Temozolomide Combined with Resveratrol on Glioma

**Figure 5:**
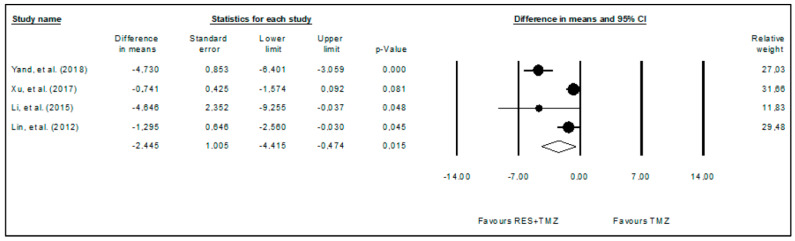
Forest plot comparing the effects of resveratrol combined with temozolomide to temozolomide alone in glioma growth. Heterogeneity: Tau^2^ = 3.011; Chi^2^ = 19.418; df = 3; *p*-value < 0.001; I^2^ = 84.551%. Test for overall effect: Z = −2.432 (*p*-value = 0.015) [9,11,27,30].

The effects of the administration of temozolomide alone compared with its administration combined with resveratrol were also subjected to a meta-analysis (Figure 5) using the random effects model to identify a potential cumulative effect between resveratrol and temozolomide. It was verified that the combination of temozolomide and resveratrol further significantly reduced (*p*-value = 0.015) glioma volume when compared to temozolomide alone. This meta-analysis included four studies comprising 30 animals (WDM = −2.445; 95%CI: −4.415 to −0.474). High heterogeneity among the included studies was also observed (I^2^ = 84.551%).

Sensitivity analysis was also performed to explore how the results would change if one or more studies were not included in this meta-analysis (Figure S1). As was the case previously, the meta-analysis of the administration of temozolomide alone compared with its administration combined with resveratrol remained unchanged, demonstrating the robustness of these findings.

The existence of publication bias was also investigated by Egger’s regression test (Table S3), and the results did not indicate its presence (*p*-value > 0.05). Considering the trim and fill approach, a funnel plot was also obtained (Figure S2), which indicated that two additional studies would be necessary to achieve an absence of publication bias.

### 3.2. Discussion

A total of 10 independent studies involving 108 animals were included in the present systematic review with meta-analysis. It was found that the resveratrol treatment was able to reduce tumor volume in murine xenografts models of glioma. The therapeutic effect of resveratrol was observed irrespective of the type of inoculated cells, human glioblastoma or rat glioma, and administration mode. Furthermore, combined treatment with resveratrol and temozolomide produced further reduction in tumor volume when compared with temozolomide alone.

Resveratrol has been shown to have an anti-tumor effect against gliomas in several prior in vitro and in vivo pre-clinical investigations using animal models [5,14,15]. To the best of our knowledge, however, this work represents the first systematic review and meta-analysis of the impact of resveratrol on glioma tumor development in animal models.

Since most of the studies reported the initial and final volume, or the fold increase in tumor volume, along with the corresponding SD or SEM, the fold increase from the initial tumor volume before treatment was chosen as the outcome analyzed in the current meta-analysis rather than the median survival time.

A subgroup analysis was performed to determine the influence of the animal model used, the type of cells inoculated, the intervention duration, the administration mode, and the mean daily dose on the effects of resveratrol on glioma growth.

Considering the site of tumor inoculation, only two studies included in the present meta-analysis used orthotopic intracranial xenografts, while most of the studies used heterotopic subcutaneous xenografts. A significant reduction in tumor volume by resveratrol treatment was only observed for the subcutaneous heterotopic xenograft model. The lack of observation of the significance of the effect of resveratrol in the orthotopic model probably resulted from the reduced number of studies using this model. It would be desirable to increase the number of studies using the orthotopic model; however, no more studies using this model were found that met the inclusion criteria. Nevertheless, there was no significant variation in the effect of resveratrol between tumor models, and the animal model used did not account for a significant proportion of the observed heterogeneity.

Regarding the type of tumor cells inoculated, most studies included in the present meta-analysis used human glioblastoma cells, only two studies used C6 [28] and RT-2 [10] rat glioma cell lines to inoculate the animals. However, a significant reduction in tumor volume produced by resveratrol administration was observed for both type of cells and the cell type did not account for the observed heterogeneity of the results.

Considering the duration of treatment with resveratrol, a significant reduction in tumor volume was found only for longer treatments (18–30 days). Furthermore, the intervention duration was shown to be a significant source of heterogeneity. Concerning the mode of resveratrol administration, all administration modes produced a significant reduction in tumor volume; however, intraperitoneal administration produced a smaller effect than either intravenous or oral administration of resveratrol. This result must be considered with caution since there was only one paper for each of the studies employing either the intravenous or oral administration of resveratrol. Nevertheless, the administration mode was a significant source of the heterogeneity.

Regarding the mean daily dose of resveratrol, a significant reduction in glioma volume was found only when higher doses (15.1–150 mg/kg) were applied. However, the mean daily dose of resveratrol did not appear to contribute significantly to the heterogeneity.

Even after subgrouping for the place of the cell tumor inoculation, the type of cells inoculated, the duration of the treatment, the route of administration, or the mean daily dose, the heterogeneity of the results in the current meta-analysis was typically moderate or high. This is common in meta-analyses utilizing data from animal models [12] since it is challenging to pinpoint the reasons for heterogeneity in these kinds of research because experimental settings vary widely. However, animal research is necessary to elucidate disease causes and assess the safety and effectiveness of therapies.

After calculating the studies’ quality ratings, it was possible to conclude that the overall quality of the studies included in the current meta-analysis is good. The results of this meta-analysis are reliable, as demonstrated by the sensitivity analysis. The present meta-analysis was evaluated for publication bias using funnel plots and Egger’s regression test. Neither the meta-analysis assessing the impact of resveratrol alone nor in combination with temozolomide demonstrated any discernible publication bias, according to the Egger’s regression test. Nonetheless, the study of funnel plots revealed the existence of publication bias, which often arises from the fact that neutral research frequently goes unreported or takes longer to publish than that presenting statistically significant findings [34]. This does not, however, negate the data from the current meta-analysis since, for both the meta-analysis assessing the impact of resveratrol alone or in combination with temozolomide, the adjusted WDM remained significant even when publication bias was considered. But as is frequently the case with animal research, it cannot be ruled out that other confounding factors from the study design, such as randomization, allocation concealment, and blinded outcome assessment, might potentially be a source of bias [35].

Given the limited chemotherapeutic alternatives available for treating gliomas, the results of this meta-analysis are interesting since they demonstrate the considerable anti-cancer impact of resveratrol against glioma and in particular glioblastoma in animal models. In a phase III clinical trial [36], the median survival time rose from 12.1 months with radiation alone to 14.6 months with radiotherapy plus temozolomide. This drug was one of the few demonstrating clinical efficacy, albeit modestly, against glioblastoma. Thus, more research should be conducted on the possible application of resveratrol, either by itself or in combination with other medications or radiation therapy, for the treatment of glioblastoma. The findings of the current meta-analysis, which demonstrated that resveratrol plus temozolomide treatment reduced tumor volume in glioblastoma-bearing animals more than temozolomide alone, raise the possibility that co-treating glioblastoma patients with temozolomide and resveratrol may improve their prognosis and also emphasize the need for more research including human clinical trials.

Several in vitro and in vivo pre-clinical studies have shown that resveratrol may produce its anti-tumor effects on glioma by acting on multiple cellular targets and modulating different signaling pathways. Resveratrol has been shown to reduce proliferation, induce apoptosis and autophagy, decreases stemness and increase differentiation, reduce epithelial-mesenchymal transition (EMT), and invasion of glioma cells as well as reducing angiogenesis in gliomas [5,7,9,10,11,14,15]. The results observed in this work, regarding the action of resveratrol in animal models of glioblastoma, are in agreement with the various studies already published on the antitumor role of this compound against glioma. In fact, resveratrol has shown the ability to reduce inflammation—which is involved in tumor initiation and progression—and has an antiproliferative effect on glioma stem cells (GSC) [5,7,15]. In addition, resveratrol decreased the activation of ERK and PI3K/Akt/mTOR pathways, resulting in decrease cell proliferation [14,15], and decreased expression of stemness markers (CD133, OCT4 and Nanog) [5,15], resulting in decreased GSC self-renewal and increased differentiation. Resveratrol also increases autophagosome formation, TP53 and p21 expression, Bax activation and Bcl-2 inhibition, thus promoting autophagy, cell-cycle arrest, and apoptosis of cancer cells while producing no significant harm against normal cells [5,15]. Furthermore, resveratrol reduces the expression of type 2 and 9 metalloproteinases (MPP-2 and 9), therefore reducing extracellular matrix degradation and invasion [14,15], and also reduces HIF1-α expression, thus decreasing angiogenesis.

In brief, resveratrol can produce its antitumor effects in glioma by modulating several pathways, either by inhibiting the stages of carcinogenesis, from the initiation to progression and invasion, or through promoting autophagy and apoptosis of cancer cells without affecting normal cells. Interestingly, in in vivo studies using animal models of glioblastoma, resveratrol increased the action of temozolomide apparently by three mechanisms: (1) via inhibition of NF-kB and consequent repression of the MGMT repair enzyme; (2) by increasing the cytotoxicity action of temozolomide by inhibiting temozolomide-induced cytoprotective autophagy; both mechanisms (1) and (2) seem to be dependent upon ROS levels reduction by resveratrol; (3) by DNA double-stranded breaks/pATM/pATR/p53 pathway activation, and by promoting glioblastoma-initiating cell differentiation involving p-STAT3 inactivation [9,11,14].

## 4. Conclusions

The present meta-analysis showed that resveratrol was effective in reducing tumor growth in animal models of glioma, particularly in heterotopic subcutaneous xenograft models. Treatment efficacy was observed either in animals inoculated with human glioblastoma or rat glioma cells and for different modes of administration of resveratrol. The efficacy of resveratrol was only evident for long-term treatment and for higher average daily doses. Furthermore, combined administration of resveratrol and temozolomide was more effective than temozolomide alone. The findings indicated publication bias, although this does not negate resveratrol’s effectiveness. These encouraging outcomes in the glioma animal model emphasize the value of resveratrol translational research, which might result in studies with practical applications.

## Figures and Tables

**Table 1:** Subgroup analysis of the effects of resveratrol on glioma growth.

Variable	Fold Increase			
	No. of Studies	WDM (95% CI)	*p*-Value	I^2^ (%)
**Animal model**				
Heterotopic	8	−2.179 (−3.707 to −0.652)	0.005 *	85.56
Orthotopic	2	−2.580 (−5.846 to 0.686)	0.122	88.79
Chi^2^ = 0.047; *p*-value = 0.828				
**Cells**				
Human	8	−1.794 (−2.917 to 0.670)	0.002 *	86.01
Animal	2	−5.678 (−10.098 to −1.258)	0.012 *	80.02
Chi^2^ = 2.786; *p*-value = 0.095				
**Intervention duration (days)**				
10–14	5	−0.954 (−1.983 to 0.075)	0.069	74.80
18–30	5	−3.778 (−5.312 to −2.243)	<0.001 *	69.36
Chi^2^ = 9.778; *p*-value = 0.008 *				
**Administration mode**				
Intraperitoneal	8	−1.140 (−2.079 to −0.200)	0.017 *	73.27
Intravenous	1	−4.272 (−8.167 to −0.377)	0.032 *	0
Oral	1	−4.404 (−6.481 to −2.327)	<0.001 *	0
Chi^2^ = 9.484; *p*-value = 0.009 *				
**Mean daily dose (mg/kg)**				
0.013–6	2	−1.340 (−4.607 to 1.327)	0.421	84.71
6.1–15	5	−1.891 (−4.217 to 0.436)	0.111	0
15.1–150	3	−3.184 (−6.596 to −1.032)	0.007 *	93.91
Chi^2^ = 1.755; *p*-value = 0.635				

WDM—weighted difference in mean; CI—confidence interval; * indicates a significant result.

**Table 2:** Assessment of publication bias for the impact of resveratrol administration on glioma growth.

Outcome	Egger’s Regression Test			
	95%CI	*t*	*p*-Value	df
Tumor volume (fold increase from day 1)	−3.839 to 1.181	1.221	0.257	8

CI—confidence interval; df—degrees of freedom.

## Data Availability

Data will be available by request to the corresponding author.

## Supplementary Materials

The following supporting information can be downloaded at: https://www.mdpi.com/article/10.3390/ijms242316597/s1.
