# Supplementary material for: Therapeutic Potential of Resveratrol for Glioma: A Systematic Review and Meta-Analysis of Animal Model Studies
Source: Int J Mol Sci. 2023 Nov 22;24(23):16597. doi: 10.3390/ijms242316597 (PMC10706392; doi:10.3390/ijms242316597)
Supplement: Supplementary file 1 [file ijms-24-16597-s001.zip › ijms-2719155-supplementary.pdf]

Table S1: Characteristics of the 10 included studies in this systematic review with meta-analysis.

Study	Year	Cells	Animals	Intervention	Outcome analyzed	Model used	Dose	Duration of the treatment	Administration mode
Yi, et al [1]	2023	U87MG-mCherry-luc	BALB/c nude female mice	Normal	Fluorescence of the tumors	Orthotopic xenografts GBM mice	-	13 days	-
				Interferon-elastin-like polypeptide (IFN-ELP(V))			1 mg IFN-equivalent/mouse		Administered into de center of the tumor
				Resveratrol (RES)			12.5 mg/kg body weight/day		Intraperitoneal injection/daily
				IFN-ELP(V) + RES			1 mg IFN-equivalent/mouse + 12.5 mg/kg body weight		Administered into de center of the tumor + Intraperitoneal injection/daily
Lin, et al [2]	2021	Rat C6 glioblastoma cell line	Male BALB/c nude mice	Normal (saline)	Tumor volume	Heterotopic (subcutaneous)	30 mg/kg/2 in 2 days	12 days	Intraperitoneal, 6 times in 2-day intervals
				Free Resveratrol					
				PP@Res Nanoparticles					
				Pep-PP@Res Nanoparticles					
Yang, et al [3]	2018	T98G (human)	Specific-pathogen-free male nude mice	Control (DMSO)	Tumor volume	Xenografts (injection in the left armpit)	-	30 days	Intraperitoneal
				Resveratrol			10 mg/kg/day		
				Temozolomide			25 mg/kg/day		
				RV + TMZ			-		
				RV + TMZ + IWR-1			plus 10 mmol/kg lithium chloride/day		
				RV + TMZ + LiCl			plus 25 mmol/kg IWR-1, the Wnt signaling pathway inhibitor/day		
				LP-resveratrol			100 µL or 300 µM		
Jhaveri, et al [4]	2018	U-87 human glioblastoma	Female athymic NCr-nu/nu nide mice	Control (PBS)	Tumor volume	Xenografts (injected subcutaneously over the left flank)	-	18 days	via the tail vein
				PL (liposomes without Resveratrol)			-		
				Free Resveratrol			6 mg resveratrol dissolved in 20% solution 2HPβCD		
				RES-L (liposomes)			10 mg/kg resveratrol equivalent in 2 days		
				Tf-RES-L (transferrin-resveratrol liposomes)			10 mg/kg resveratrol equivalent		
Xu, et al [5]	2017	U87	Nude mice	Control	Relative tumor volume	Xenografts (injected subcutaneously into the left axillary space)	-	14 days	Intraperitoneal
				Resveratrol			10 mg/kg		
				Temozolomide			30 mg/kg		
				Temozolomide + Resveratrol			Equivalent dose		
				T/R-NPs			Equivalent dose		
				Resveratrol			100 µL or 300 µM		
Clark, et al [6]	2017	U87 glioma cells	Female BALB/c nude mice	Control (vehicle)	Tumor size	Xenografts (subcutaneous injection)	-	18 days	Oral
				Resveratrol			water containing 0.1 mg/mL resveratrol ad libitum		
Wang, et al [7]	2015	Human GSC line SU-2	Male nude BALB/c mice	Control (saline solution)	Relative tumor volume		-	14 days	day 3 and day 9 with a dose rate of 2 Gy/min; Intraperitoneal injection/day
				X-ray			6 Gy		

				Resveratrol		Xenografts (implanted subcutaneously)	150 mg/kg/day		
				X-ray + Resveratrol			6 Gy + 150 mg/kg		
Li, et al [8]	2015	Human glioblastoma initiating cells	Female NOD/SCID mice	Control	Tumor volume	Xenografts (injected subcutaneously into the left hind flank)	-	30 days	oral gavage; resveratrol injected intraperitoneally
				Temozolomide			68 mg/kg		
				Resveratrol			12.5 mg/kg/day		
				Temozolomide + Resveratrol			Equivalent doses		
				Curcumin			50 mg/kg	14 days	Intraperitoneal injection
				Resveratrol			10 mg/kg/day	-	
				Temozolomide			10 mg/kg/3 per week	-	
				Temozolomide + Curcumin			Equivalent doses	-	
				Temozolomide + Resveratrol			Equivalent doses	-	
				Temozolomide + Curcumin + Resveratrol			Equivalent doses	-	
				Temozolomide + Chloroquine			Equivalent doses + 20 mg/kg	-	
				Temozolomide + Curcumin + Chloroquine			Equivalent doses	-	
				Chloroquine			Equivalent doses	-	
				Lipid-core nanocapsules (LNC)			-	-	
				Resveratrol			5 mg/kg/day	-	
				Resveratrol-LNC			5 mg/kg/day	-	
				Resveratrol			40 mg/kg	-	
				Temozolomide			68 mg/kg		
				Resveratrol + Temozolomide			Equivalent doses		
Lin, et al [9]	2012	U87	Female BALB/c nude mice	Control (vehicle)	Tumor volume	Xenografts (subcutaneously injected into the right hind flank of the mice)	10% DMSO	12 days	Intraperitoneal injection
				Temozolomide			10 mg/kg/day		
				Resveratrol			12.5 mg/kg/day		
				Temozolomide + Resveratrol			Equivalent doses		
				CD133+/Sh-Scramble			-		
				CD133+/Resveratrol			-		
				CD133+/Sh-STAT3			-		
				CD133+/Sh-STAT3+Resveratrol			-		
Tseng, et al [10]	2004	rat RT-2 glioma cell line	Fischer 344 rats	Control	Tumor volume	Orthotopic (injected into the right caudate-putamen)	-	28 days	Intraperitoneal injection
				Vehicle			-		
				Resveratrol			40 mg/kg/day		

Table S2: Study quality scores.

Study	Year	1	2	3	4	5	6	7	8	9	Quality score
Yi, et al [1]	2023	+	+	+	-	-	+	+	+	-	6
Lin, et al [2]	2021	+	+	+	-	-	+	+	+	-	6
Yang, et al [3]	2018	+	+	+	-	-	+	+	-	-	5
Jhaveri, et al [4]	2018	+	+	+	-	-	+	-	+	-	5
Xu, et al [5]	2017	+	+	-	-	-	+	+	-	-	4
Clark, et al [6]	2017	+	+	+	-	-	+	-	+	-	5
Wang, et al [7]	2015	+	+	+	-	-	+	+	+	-	6
Li, et al [8]	2015	+	+	-	-	-	+	-	+	-	4
Lin, et al [9]	2012	+	-	+	-	-	-	-	+	-	3
Tseng, et al [10]	2004	+	+	-	-	-	-	-	+	-	3

1) Peer-review publication;
2) Standardized number of tumor cells implanted;
3) Randomized allocation of tumor-bearing animals to treatment and control groups;
4) Blinded assessment of outcome;
5) Sample size calculation performed;
6) Compliance with animal welfare regulations;
7) Statement of potential conflicts of interest;
8) Reported the number of animals originally inoculated with tumor cells;
9) Reported the explanation of any treated animals excluded from analysis.

Table S3. Assessment of publication bias for the impact of administration of temozolomide combined with resveratrol on glioma growth.

Outcome	Egger's regression test			
	**95%CI**	***t***	***p*-value**	**df**
Tumor volume (fold increase from day 1)	-13.896 to 6.914	1.444	0.286	2

CI – confidence interval; df – degrees of freedom.

Figure S1: Results of sensitivity analysis for the meta-analysis of the of administration of temozolomide combined with resveratrol on glioma growth.

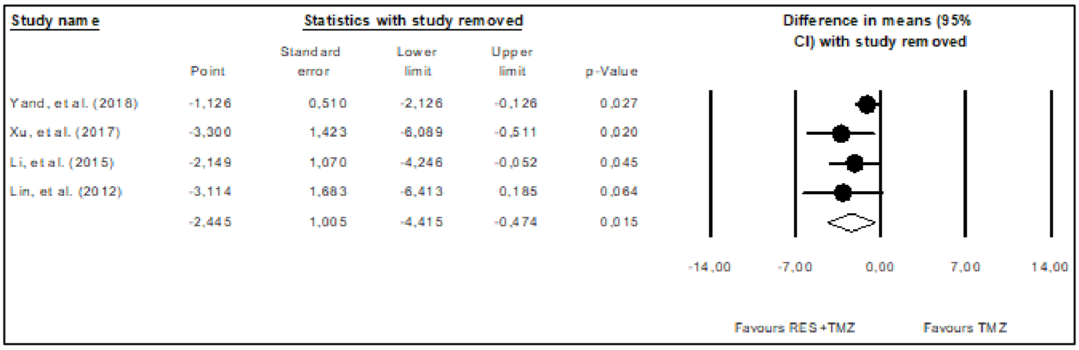

Figure S2: Funnel plot of standard error by difference in means (publication bias tests) of the effects of administration of temozolomide combined with resveratrol on glioma growth.

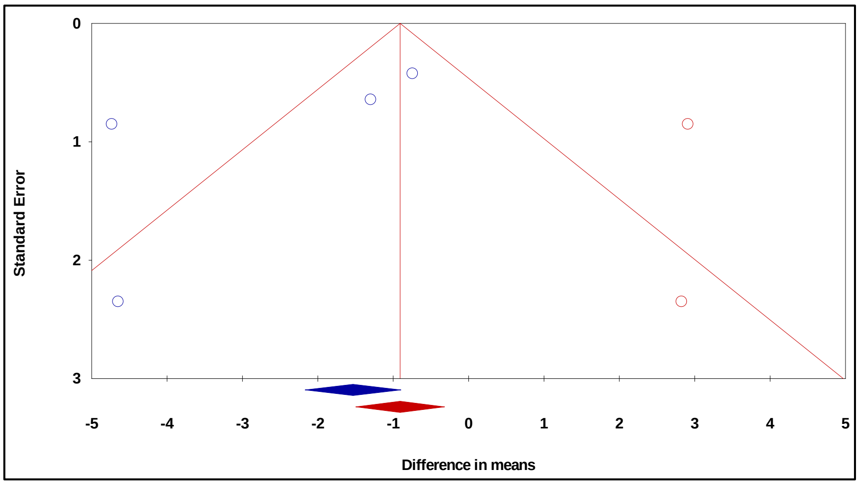

References

1. Yi, G.; Liu, H.; Sun, F.; Du, R.; Kong, J.; Wang, H.; Cheng, H.; Wang, G.; Gao, F.; Liang, P. Intratumor Injection of Thermosensitive Polypeptide with Resveratrol Inhibits Glioblastoma Growth. *Tissue Eng. Part C Methods* **2023**, *29*, 103–109. https://doi.org/10.1089/ten.tec.2022.0207.
2. Lin, X.; Shi, X.; Xiong, L.; Nie, J.; Ye, H.; Du, J.; Liu, J. Construction of il-13 receptor α2-targeting resveratrol nanoparticles against glioblastoma cells: Therapeutic efficacy and molecular effects. *Int. J. Mol. Sci.* **2021**, *22*, 10622. https://doi.org/10.3390/ijms221910622.
3. Yang, H.-C.; Wang, J.-Y.; Bu, X.-Y.; Yang, B.; Wang, B.-Q.; Hu, S.; Yan, Z.-Y.; Gao, Y.-S.; Han, S.-Y.; Qu, M.-Q. Resveratrol restores sensitivity of glioma cells to temozolamide through inhibiting the activation of Wnt signaling pathway. *J. Cell. Physiol.* **2019**, *234*, 6783–6800. https://doi.org/10.1002/jcp.27409.
4. Jhaveri, A.; Deshpande, P.; Pattni, B.; Torchilin, V. Transferrin-targeted, resveratrol-loaded liposoms for the treatment of glioblastoma. *J. Control. Release* **2018**, *277*, 89–101. https://doi.org/10.1016/j.jconrel.2018.03.006.
5. Xu, H.; Jia, F.; Singh, P.K.; Ruan, S.; Zhang, H.; Li, X. Synergistic anti-glioma effect of a coloaded nano-drug delivery system. *Int. J. Nanomed.* **2017**, *12*, 29–40. https://doi.org/10.2147/IJN.S116367.
6. Clark, P.A.; Bhattacharya, S.; Elmayan, A.; Darjatmoko, S.R.; Thuro, B.A.; Yan, M.B.; van Ginkel, P.R.; Polans, A.S.; Kuo, J.S. Resveratrol targets AKT and p53 in glioblastoma and glioblastoma stem-like cells to suppress growth and infiltration. *J. Neurosurg.* **2017**, *126*, 1448–1460. https://doi.org/10.3171/2016.1.JNS152077.
7. Wang, L.; Long, L.; Wang, W.; Liang, Z. Resveratrol, a potential radiation sensitizer for glioma stem cells both in vitro and in vivo. *J. Pharmacol. Sci.* **2015**, *129*, 216–225. https://doi.org/10.1016/j.jphs.2015.11.001.
8. Li, H.; Liu, Y.; Jiao, Y.; Guo, A.; Xu, X.; Qu, X.; Wang, S.; Zhao, J.; Li, Y.; Cao, Y. Resveratrol sensitizes glioblastoma-initiating cells to temozolomide by inducing cell apoptosis and promoting differentiation. *Oncol. Rep.* **2016**, *35*, 343–351. https://doi.org/10.3892/or.2015.4346.
9. Lin, C.J.; Lee, C.; Shih, Y.; Lin, T.; Wang, S.; Lin, Y.; Shih, C. Resveratrol enhances the therapeutic effect of temozolomide against malignant glioma in vitro and in vivo by inhibiting autophagy. *Free Radic. Biol. Med.* **2012**, *52*, 377–391. https://doi.org/10.1016/j.freeradbiomed.2011.10.487.
10. Tseng, S.; Lin, S.; Chen, J.; Su, Y.; Huang, H.; Chen, C.; Lin, P.; Chen, Y. Resveratrol Suppresses the Angiogenesis and Tumor Growth of Gliomas in Rats. *Crit. Cancer Res.* **2004**, *10*, 2190–2205. https://doi.org/10.1158/1078-0432.ccr-03-0105.
